# Infusion of Trx-1-Overexpressing hucMSC Prolongs the Survival of Acutely Irradiated NOD/SCID Mice by Decreasing Excessive Inflammatory Injury

**DOI:** 10.1371/journal.pone.0078227

**Published:** 2013-11-04

**Authors:** JiangWei Hu, ZaiLiang Yang, Jun Wang, YongYong Tang, Hao Liu, Bin Zhang, Hu Chen

**Affiliations:** 1 Department of Hematopoietic Stem Cell Transplantation and Gene Therapy Center, Affiliated Hospital of Academy of Military Medical Sciences, Beijing, China; 2 Department 4, State Key Laboratory of Trauma, Burn and Combined Injury, Research Institute of Surgery and Daping Hospital, Third Military Medical University, Chongqing, China; National Institutes of Health, United States of America

## Abstract

A protective reagent for ARI should have the ability to repair injured tissue caused by radiation and prevent continuous damage from secondary risk factors. Trx-1 was explored as a candidate therapy for ARI, as it scavenges reactive oxygen species, regulates cell growth and differentiation, participates in immune reactions, and inhibits apoptosis by acting inside and/or outside cells. Trx-1 can also decrease excessive inflammation in ARI by regulating the creation of inflamed media, by inhibiting the activation of complement, and by reducing the chemotaxis, adhesion, and migration of inflammatory cells. As effectively and stably expressing exogenous genes in the long term and regulating immune inflammation and tissue repair, MSC are a good choice for Trx-1 gene therapy. In this study, Trx-1-overexpressing hucMSC-Trx-1 were obtained by adenoviral vector-mediated infection. We first measured the redox capacity of hucMSC-Trx-1 with an antioxidant capacity (T-AOC) assay, a hydrogen peroxide (H_2_O_2_) content determination assay *in vivo*, a H_2_O_2_-induced oxidation hemolysis assay, and a lipid peroxidation assay *in vitro*. Then, we measured survival time, the protection of the hematopoietic system, and the regulation of inflammation in important organs in three treatment groups of NOD/SCID mice (treated with hucMSC-Trx-1, with hucMSC, and with saline) that were exposed to 4.5 Gy ^60^Co-γ-ray radiation. The hucMSC-Trx-1 group achieved superior antioxidation results, protecting bone marrow hematopoietic stem cells (Lin^−^CD117^+^: hucMSC-Trx-1 *vs*. hucMSC, *P*<0.05; hucMSC-Trx-1 *vs*. NS, *P*<0.01), promoting the formation of red blood cells and hemoglobin (hucMSC-Trx-1 *vs*. hucMSC or NS, *P*<0.05), reducing inflammation and damage in important organs (Bone marrow and lung: hucMSC-Trx-1 *vs*. NS, *P*<0.01; hucMSC-Trx-1 *vs*. hucMSC, *P*<0.05. Liver and intestine: hucMSC-Trx-1 *vs*. NS, *P*<0.05; hucMSC-Trx-1 *vs*. hucMSC, *P*<0.05), and prolonging survival (hucMSC-Trx-1 *vs*. hucMSC or NS, *P*<0.01). Therefore, hucMSC-Trx-1 combines the merits of gene and cell therapy as a multifunctional radioprotector for ARI.

## Introduction

As technology progresses, radioactive materials are increasingly used in medicine, electricity, mining exploration, and many different types of research. While radioactivity has many beneficial uses, it is also dangerous. A common type of radiation-related harm, acute radiation injury (ARI), is characterized by its intense nature, rapid progression, and poor prognosis; the incidence of ARI is increasing [1]–[3]. Therefore, ARI has become a focus of radiation prevention research.

In the past, the strategy for the prevention and treatment of ARI mainly focused on improving the status of bone marrow transplantation, improving hemopoiesis, and treating complications. However, ARI is a whole-body, multi-organ and multi-issue injury caused by a large dose of ionizing radiation (γ-ray, X-ray or neutron) over a short period of time. Its pathology is a gradually evolving, complicated process. It not only includes direct damage from radioactive material but also indirect continuing damage caused by the generation of a series of active materials and over-inflammation. Finally, the decompensate inflammation of organs and ensuing complications further affect the health and mortality of radiation victims.

Currently, the prevention and treatment of ARI involves chemical medicines (e.g., WR-2721, CBLB-502), natural medicines [4], [5], biological reagents, and small molecules [6], [7]. These reagents are used to protect sulfhydryls of target molecules, reduce free radicals, and stabilize the DNA structure; however, they have disadvantages such as a short half-life, an easily degradable nature, and the ability to cause serious adverse events, making them sub-optimal for clinical use. In addition, the growth factors [8], which have been used for ARI, only facilitate committed stem/progenitor cell multiplication and acceleration, and few are able to affect pluripotent stem cells. Although growth factors lead to a quick increase in peripheral blood cells, they also lead to the exhaustion of hematopoietic stem cells (HSC) after radiation, which increases the difficulty of hematopoietic recovery. The transplantation of HSC offers a new approach for treating ARI; however, the intensified pretreatment and the application of immunosuppressants can intensify the radiation-induced failure of numerous organs, with the result that HSC [9] transplantation has not significantly decreased total mortality.

Therefore, a new effective treatment based on the mechanisms of the pathogenesis of ARI is needed. Our research focuses on the protection of important organs and the promotion of their functional recovery after radiation injury.

With a molecular mass of 12 kDa, Thioredoxin (Trx) is found in the cytoplasm of many bio-organism eukaryotic organisms [10]; the active site of this molecule is Cys-Gly-Pro-Cys. The redox capacity of Trx originates from its ability to combine with substrate X-S2 to reduce protein substrates. Therefore, if two cysteines in the activation center are mutated to Ser (C32S/C35S), the reductive activity of this molecule is lost [11]. Because it is a disulfide reductase containing a conserved catalytic site, Trx modifies a series of key enzymes and other important substances by altering the oxidation and reduction states in the body [12], [13]. Trx is different from other antioxidants such as SOD and N-acetylcysteine, because Trx not only regulates the redox balance in cells by scavenging intracellular peroxide hydrogen, oxygen free radicals and other ROS ingredients, but it also has a variety of biological activities, including the regulation of cell growth, transcription factors [14], [15], gene expression, apoptosis [16], [17], and immunoregulatory effects [18]–[20]. Of a variety of known subtypes of Trx, Thioredoxin-1 (Trx-1) has been chosen for use in exogenous gene therapy because of its existence in mammalian somatic cell cytoplasm and its extracellular secretion [21], [22]. In human plasma and serum, the concentration of Trx-1 is 10–30 ng/ml; in patients with oxidative stress, its circulating concentration is 40–140 ng/ml, and its tissue/organ level is 0.1–10 µg/ml [23]. Secreted Trx-1 may facilitate cell growth. This facilitation depends on the redox capacity of this catalyst [24]. Moreover, extracellular Trx-1 can inhibit cell damage or apoptosis induced by H_2_O_2_ [25]. This protection likely occurs through the interaction of Trx-1 with target molecules in the cell membrane; alternatively, Trx-1 might re-enter the cell to perform this function [26]. Meanwhile, high-concentration Trx-1 not only inhibits LPS-induced IL-1β expression and secretion, it also inhibits granulocyte and monocyte tropism to inflammation loci. In addition, Trx-1 can regulate the transduction of inflammation signals and cell adhesion and thereby prevent over-inflammation. However, as a biologically active factor, Trx-1 is easily diluted in the blood and rapidly degraded when it enters the body and therefore cannot reach injured regions to function continuously. Thus, finding a suitable carrier to deliver Trx-1 and to promote Trx-1 gene expression is the major challenge of developing a therapeutic form of Trx-1 that functions continuously.

Because Mesenchymal stem cells (MSC) appear to have many functions [27]–[30] (chemotaxis, migrating and homing to injury sites, secretion of hematopoietic growth factors, improvement of the hematopoietic microenvironment to support hematopoiesis, immune regulation to mitigate locally excessive inflammatory responses, and tissue repair), the potential applications of MSC for the treatment of radiation injuries have been well recognized. Compared with other MSC, human umbilical cord-derived mesenchymal stem cells (hucMSC) are more abundant, easier and less expensive to collect, more malleable, and easier to use for exogenous gene transfection and expression; as a result, hucMSC are an ideal carrier for gene therapy [31]–[33].

In this context, we used Trx-1-overexpressing hucMSC to treat NOD/SCID mice with ARI. The purpose of this study was to evaluate the efficacy of the protective treatment of ARI-exposed NOD/SCID mice with hucMSC-Trx-1 and to explore the relationship between excessive inflammation and the damage produced by ARI. By combining the functions of Trx-1 and MSC, we provide reliable experimental evidence for the value of combination gene and cell therapy for ARI.

## Materials and Methods

### Construction of the adenoviral expression vector Ad-Trx-1-EGFP

Total mRNA of hucMSC was extracted by Trizol then reverse-transcribed into cDNA. A pair of Trx-1 primers, one with a *NotI* site and one with an *EcoRV* site, was designed according to the coding sequence (NM003329) in GenBank (primers: 5′-GCCGCGGCCGCATGGTGAAGCAGATCGAG-3′, 5′-CTGGATATCTTAGACTAATTCATTAATGGTGG) -3′.

The target gene (Trx-1) was obtained from cDNA by PCR (Invitrogen, Carlsbad, CA, USA). Then, the restriction enzyme digestion product of the T vector (Invitrogen, Carlsbad, CA, USA) containing Trx-1 and the adenovirus shuttle vector pDC316-mCMV-EGFP were ligated into pDC316-TRX-1-EGFP by T4 DNA ligase (TaKaRa, Otsu Japan). Subsequently, pDC316-hTRX-EGFP was linearized by *PmeI*, transformed into competent *Escherichia coli* DH-5α cells, amplified, extracted, and purified (TaKaRa, Otsu Japan). The expressed product of pDC316-TRX-1-EGFP was identified by sequencing. Finally, the shuttle plasmid (pDC316-Trx-1-EGFP) and backbone plasmid (pBHGloxΔE1, 3Cre) were cotransfected into HEK293 cells by Lipofectamine™2000 (Invitrogen, Carlsbad, CA, USA) to obtain the adenoviral expression vector Ad-Trx-1-EGFP. Ad-Trx-1-EGFP was packaged, amplified, and purified. The titer and number of particles were determined with the TCID50 method.

### Collection and Identification of hucMSC

The umbilical cord was obtained from a term infant who was born through natural childbirth at the Department of Obstetrics, General Hospital of the People's Liberation Army, Beijing, China. Under sterile conditions, it was rinsed with phosphate-buffered saline (PBS) and cut into 1–2 mm^3^ pieces. These tissues were digested for one hour with 0.1% collagenase II (Sigma, St. Louis, MO, USA) in a water bath at 37°C. The product of digestion was filtered through a 100-mesh strainer and centrifuged. The precipitate of cells was suspended in DMEM/F12 (HyClone, Logan, UT, USA) complete culture medium (100 IU/ml penicillin, 100 µg/ml streptomycin and 10% FBS). Resuspended cells were plated at a density of 1×10^5^/cm^2^ and cultured at 37°C in 5% CO_2_ and saturated humidity. The medium was changed every third day. The adherent cells were subcultured at a proportion of 1:3 when the cells reached 80% confluence. Cell morphology and growth were observed by microscopy. The viability of cells was detected by trypan blue staining [34].

Flow cytometry was used to identify the immune phenotype of third-generation hucMSC, which were labeled with monoclonal antibodies against CD105-PE, CD73-PE, CD31-PE, CD166-PE, CD34-PE, CD80-PE, CD14-FITC, CD86-FITC, CD90-FITC, HLA-ABC-FITC, HLA-DR-FITC, CD45-FITC APC, or isotype control (BD Biosciences, San Diego, CA, USA). The DNA content of hucMSCs incubated with propidium iodide (PI, final concentration 50 µg/ml) (Sigma Amresco, St. Louis, MO, USA) was detected by flow cytometry (Coulter EPICS XL, BD Biosciences, San Diego, CA, USA), and the results were analyzed with ModiFIT software to identify the cell cycle.

Third-generation hucMSC were resuspended and plated in six-well plates at a density of 4×10^4^ cells per well to induce adipogenesis and osteogenesis. At the same time, other third-generation hucMSC at a density of 3×10^5^ cells per tube were centrifuged and incubated in 10-ml centrifuge tubes to induce chondrogenesis.

The cells were cultured in specific induction media. The basal medium consisted of DMEM/F12 and 10% FBS; adipogenic induction medium consisted of basal medium supplemented with 0.5 mM isobutylmethylxanthine (IBMX) (Sigma-Aldrich, St. Louis, MO, USA), 0.1 µM dexamethasone, 0.1% insulin, and 0.1 mM indomethacin (BD Biosciences, San Diego, CA, USA); osteogenic induction medium consisted of basal medium supplemented with 0.1 µM dexamethasone, 0.05 mM ascorbate-2-phosphate, 10 mM β-glycerolphosphate (Sigma-Aldrich, St. Louis, MO, USA), and 1% insulin; chondrogenic induction medium consisted of basal medium supplemented with 0.1 µM dexamethasone, 0.05 mM ascorbate-2-phosphate, 1 mM sodium pyruvate, 10 ng/ml TGF-β3, and 1× ITS+1 (Sigma-Aldrich, St. Louis, MO, USA). Two weeks later, the differentiation of adipocytes was analyzed by oil red O staining (Sigma-Aldrich, St. Louis, MO, USA). Osteoblastogenesis and chondrogenesis was detected with von Kossa staining and toluidine blue staining (Sigma Amresco, St. Louis, MO, USA) three weeks after induction.

### The construction and detection of hucMSC-Trx-1

Third-generation hucMSC were infected with the adenoviral expression vector Ad-Trx-1-EGFP with different multiplicities of infection (MOIs): 0, 10, 50, 100, 150, 200, 250, and 350 pfu/cell. Two hours later, hucMSC were transferred to complete culture medium and incubated for 48 hours. The Trx-1 expression efficiency of infected cells was detected by flow cytometry and fluorescence microscopy.

Forty-eight hours after infection, the expression of Trx-1 was detected by real-time quantitative reverse transcription–PCR (qRT-PCR) (Trx-1 primers: 5′-GCCTTTCTTTCATTCCCTC-3′, 5′-TTCACCCACCTTTTGTCC-3′; β-actin Primers: 5′-TGAAGGTCGGAGTCAACGG-3′, 5′-TGGAAGATGGTGATGGGATT

-3′). Briefly, the RNA of hucMSC and hucMSC-Trx-1 was extracted with TRIzol (Invitrogen, Carlsbad, CA, USA) and reverse-transcribed into cDNA with RevertAid™ M-MulV Reverse Transcriptase (Invitrogen, Carlsbad, CA, USA). Using the cDNA as a template and the SYBR® Green Realtime PCR Master Mix (Toyobo, Tokyo, Japan), we amplified genes of Trx-1 and β-actin. With the 2^−ΔΔCt^ method, we compared the difference in relative expression of the Trx-1 gene between hucMSCs and hucMSC-Trx-1.

At the same time, the protein expression of Trx-1 was detected by western blot (rabbit anti-human Trx-1 antibody (Abcam, Cambridge, UK); rabbit anti-human β-actin antibody (Cell Signaling, Danvers, MA, USA). First, cells were lysed in RIPA buffer containing 50 mM Tris/HCl (pH 8.0), 150 mM NaCl, 1% Nonidet-P40, 1% sodium deoxycholate, 0.1% SDS, 0.1 mM DTT, 0.05 mM PMSF, 0.002 mg/mL aprotinin, 0.002 mg/mL leupeptin, and 1 mM NaVO_3_. The total protein was isolated with a commercial extraction kit (Pierce, Rockford, IL, USA). Equal amounts of protein were separated by 10% SDS-PAGE and transferred onto polyvinylidene difluoride membranes. The membranes were incubated overnight with appropriate primary antibodies. Bound antibodies were then visualized using alkaline phosphatase-conjugated secondary antibodies. We used β-actin as the loading control for the Trx-1 protein.

The identification of immune phenotypes and cell cycle stages and the differentiation of hucMSC-Trx-1 was performed as described above.

### Redox capacity of hucMSC-Trx-1

Male NOD/SCID mice (weighing 23.0±1.0 g) were randomized into four groups of six. The normal control group received no treatment, while the mice of the saline group, the hucMSC group, and the hucMSC-Trx-1 group were injected intravenously with 0.2 ml of saline, 1×10^6^ normal cultured hucMSC suspended in 0.2 ml of saline, and hucMSC-Trx-1 suspended in 0.2 ml of normal saline, respectively. After 48 hours, peripheral blood was obtained through the orbit from removed eyeballs and centrifuged for 10 minutes at 3500 rpm; plasma and red blood cells were collected. Lung and liver tissue was prepared into a 10% homogenate by NS. The protein concentration in each sample was measured by bicinchoninic acid (BCA) assay.

The T-AOC values of the plasma and the lung and liver tissue homogenate were detected with a commercial kit (Jiancheng Bioengineering Institute, Nanjing, China). This kit uses antioxidants in the samples to reduce Fe^3+^ to Fe^2+^, which is then chelated with porphyrin to produce a purple complex. We quantified this complex by measuring the absorbance at 520 nm. The relative T-AOC values of the samples were normalized to the units of plasma or the protein concentration in the lung or liver tissue. H_2_O_2_ was measured with a hydrogen peroxide assay kit (Jiancheng Bioengineering Institute, Nanjing, China). The principle of the assay is to generate a stable chelating material through the interaction between H_2_O_2_ and molybdic acid. We measured the OD of this material at 405 nm. By comparing sample values with those of the standard, we obtained the exact concentration of H_2_O_2_. First, the 0.1 ml sample and 1 ml of schizolysis solution supplied by the kit were mixed and incubated at 37°C for one minute. Finally, we mixed the molybdate reagent until it had sufficient reaction. Measured the concentration of the product with a spectrometer at a wavelength of 405 nm.

We collected sedimented erythrocytes and washed them three times with NS. Then, we added 0.05 ml of the sediment to 10 ml of saline to prepare a 0.5% erythrocyte suspension. These erythrocyte samples were separated into four groups: the control (n = 6) samples contained 0.8 ml of erythrocyte suspension and 0.2 ml of saline and were incubated at 37°C for two hours; the other three groups (n = 6 each) consisted of 0.8 ml of erythrocyte suspension and 0.2 ml of saline (H_2_O_2_ control group), 1×10^6^ normal cultured hucMSC in 0.2 ml of saline, or 1×10^6^ hucMSC-Trx-1 in 0.2 ml of saline; these solutions were incubated for one hour at 37°C; then H_2_O_2_ (Sigma-Aldrich, St. Louis, MO, USA) was added at a final concentration of 300 µM and the solutions were further incubated for one hour. The samples were diluted five times in NS and centrifuged (3000 rpm, 10 min). The supernatant absorbance was measured at 415 nm. Hematolysis was calculated with the formula: Hematolysis (%)  =  A (treatment group) / A (model control).

This experiment consisted of two sections: auto-oxidation and H_2_O_2_-induced oxidation. From each section, liver tissue homogenates were collected. As in experiment 4.1, the mice were divided into four groups (n = 6 per group), the necessary experimental material was added, and the samples were incubated for one hour at 37°C. Subsequently, the liver homogenates of the auto-oxidation group continued incubating for one hour under the same conditions, while the H_2_O_2_-induced oxidation group was incubated in H_2_O_2_ (Sigma-Aldrich, St. Louis, MO, USA) at a final concentration of 300 µM for one hour. Last, we used a commercial kit (Beyotime Biotechnology, Nantong, China) to quantify the amount of malondialdehyde (MDA) generated according to the manufacturer's protocol. In brief, we added 1 ml of 15% trichloroacetic acid to terminate the reaction and 1 ml of 0.67% TBA and heated the sample to 95°C; the sample was then cooled and centrifuged. The supernatant absorbance was measured at 532 nm. By comparing the concentration of the sample with that of the manufacturer's standard, we obtained the content of MDA.

### The function of hucMSC- Trx-1-EGFP in ARI

Male NOD/SCID mice (weighing 23.0±1.0 g) were selected randomly and fed in a sterile environment. They received drinking water with antibiotics (0.1 g/L ciprofloxacin) from three days before treatment to thirty days after treatment; they were then fasted six hours before irradiation. The mice were irradiated with ^60^Co-γ-ray (total dose: 4.5 Gy; source distance: 80 cm; dose rate: 1.60 Gy/min) in a stretched position.

Mice were randomly divided into three experimental groups. Three hours after irradiation, the interrelated treatments (as follows) were given through tail vein injection under sterile conditions: the NS control group was treated with 0.2 ml of normal saline; the hucMSC group was treated with 1×10^6^ normal cultured hucMSC suspended in 0.2 ml of normal saline; and the hucMSC-Trx-1 group was treated with 1×10^6^ highly Trx-1-expressing hucMSC with the same dose used in hucMSC group.

The day of irradiation was set as day 0. Animals (n = 12 in each group) were observed daily for survival, change in body weight and change in activity. The interrelated data were analyzed after the experiment.

Twenty microliters of tail vein blood from each mouse (n = 6 in each group) were collected at different times (1 d, 4 d, 7 d, 11 d, 20 d and 30 d) in tubes with EDTA anticoagulant to analyze the dynamic changes of whole blood cells (XS-800i, Sysmex).

Bone marrow samples from each group were collected in PBS containing 0.5% NaN_3_ on day 30, and were analyzed by flow cytometry with CD117 and Lin antibodies (Biolegend, San Diego,CA, USA).

Mice (n  =  6) selected randomly from each group were sacrificed on day 30. Tissues of the femur, lung, liver, and intestine were collected for HE staining. We observed the differences in histology and scored the degree of inflammation in pathological sections of the bone marrow, lung, liver, and intestine with methods introduced by Justesen [35], Underwood [36], Ishak [37], and Obermeier [38]. The histological score was estimated by two independent investigators blinded to the treatment. In brief, (1) the degree of damage to bone marrow was evaluated by dividing one section into 360 points (36 points in 10 microscopic fields) using a computer-assisted graph collecting system; the hematopoietic tissue volume fraction per total volume fraction (HV/TV) was quantified. The total volume fraction (TV) refers to the sum of AV, HV and BV. (2) Lung inflammation was scored with standard grades from 0 (low) to five points (high inflammation). Three factors were evaluated for each section: perivascular and peribronchiolar eosinophilia, edema, and epithelial damage. Finally, we summed the scores of the three aspects to obtain the total score. A higher total score indicated a higher degree of inflammation. (3) Liver inflammation was scored with standard grades from 0 to 18 points. Four aspects were evaluated: portal area inflammation (0-4 points), piecemeal necrosis (0-4 points), spotty necrosis and focal inflammation (0-4 points), and bridging and/or confluent necrosis (0-6 points). A higher total score indicated a higher degree of inflammation. (4) Intestinal inflammation was scored with standard grades from 0 to 8 points; two aspects were evaluated: injury of the epithelium (0-4 points) and infiltration of inflammatory cells (0-4 points). A higher total score indicated a higher degree of inflammation.

### Statistical analysis

All of the data are presented as the mean ± standard error (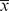 ±s). Data were processed with the statistical software SPSS version 13.0. The t-test and analysis of variance (ANOVA) were applied to test the difference between groups. A p-value of p<0.05 was considered to indicate a significant difference.

### Ethics Statement

This study was approved by the Institutional Review Board of the Affiliated Hospital of the Academy of Military Medical Sciences (protocol #2010-05-60), Beijing. A Written Informed Consent Form was obtained from the healthy umbilical cord donor. The experiments were carried out in accordance with the guidelines of current Chinese legislation for the use and care of laboratory animals. All animal treatment procedures were approved by the Institutional Animal Care and Use Committee of the Academy of Military Medical Sciences. Mice were provided with pathogen-free water and food for maintenance and caged in a controlled SPF environment with a 12/12-h light/dark cycle.

## Results

### Obtaining the high-titer recombinant adenovirus Ad-Trx-1-EGFP

Eight days after the adenovirus Ad-Trx-1-EGFP infection of 293 cells, highly increased expression of EGFP virus plaque CPE was detected. The retrieved virus DNA was amplified by PCR. After repeated infections, amplifications, and purifications, the virus titer of adenovirus Ad-Trx-1-EGFP was 5.558×10^10^ pfu/ml.

### Ad-Trx-1-EGFP-infected hucMSC shows high Trx-1 expression under optimum conditions

**Figure 1:**
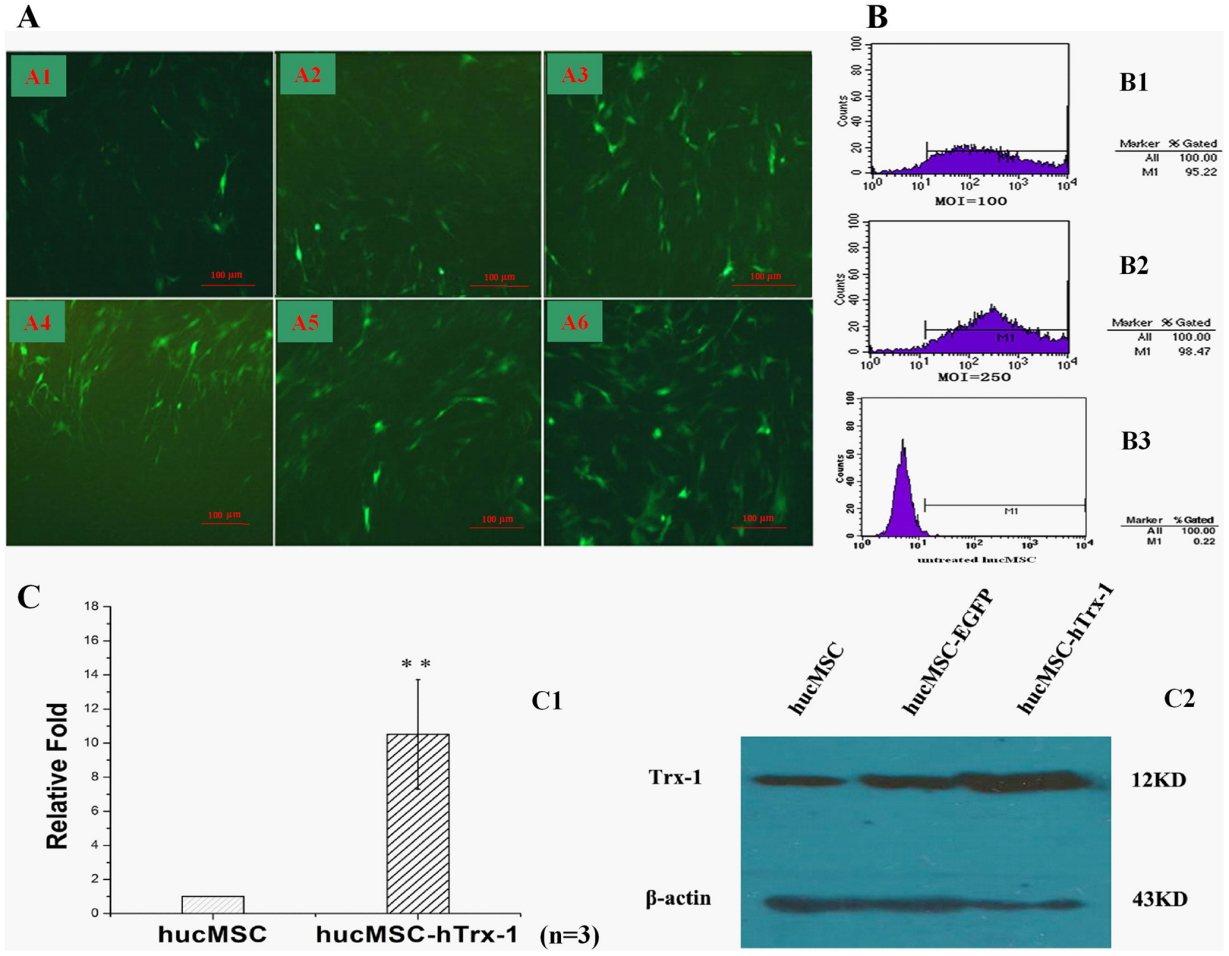
The hucMSC's optimum condition for gene modification with adenovirus-Trx-1 and the detection of Trx-1 gene expression in hucMSC-Trx-1. The following detections were performed in 48-Trx-1-EGFP infection. (A) Infection efficiency of hucMSC-Trx-1 was detected by EGFP using fluorescence microscope (A1: MOI = 10; A2: MOI = 50; A3: MOI = 100; A4: MOI = 150; A5: MOI = 200; A6: MOI = 250. ×100). (B) Compared with MOI = 100 (B1), infection efficiency of MOI = 250 (B2) showed no significant increase in hucMSC-Trx-1 by flow cytometry, however the untreated hucMSC has scarcely any positive expression of fluorescent signal (B3). (C) The mRNA expression of Trx-1 gene in hucMSC-Trx-1 was increased 10.52±3.21 fold compared to hucMSC by fluorescent quantitative RT-PCR (^**^ *P*<0.01). (D) The Trx-1 protein expression in hucMSC-Trx-1, hucMSC and hucMSC-EGFP were detected by Western blotting.

Forty-eight hours after virus infection, flow cytometry indicated that the efficiency of hucMSC Ad-Trx-1-EGFP infection increased with increasing MOI. However, when the MOI exceeded 100, the infection efficiency stopped increasing, and MOI values of 200 or more led to cell growth deterioration. When the MOI was greater than 250, cell death was detected. These results were confirmed by fluorescence microscopy. The MOI value of 100 was chosen as the optimal condition for infection (Fig. 1 A, B).

Adenoviral infection was performed with the MOI value of 100, and hucMSC-Trx-1 transcription was analyzed by fluorescent quantitative RT-PCR (sq-PCR). In hucMSC-Trx-1, Trx-1 expression was 10.52±3.21-fold higher than in the control group (Fig. 1 C). The western blot results confirmed this increase at the protein level (Fig. 1 D).

### hucMSC-Trx-1 retain their classic MSC characteristics

**Figure 2:**
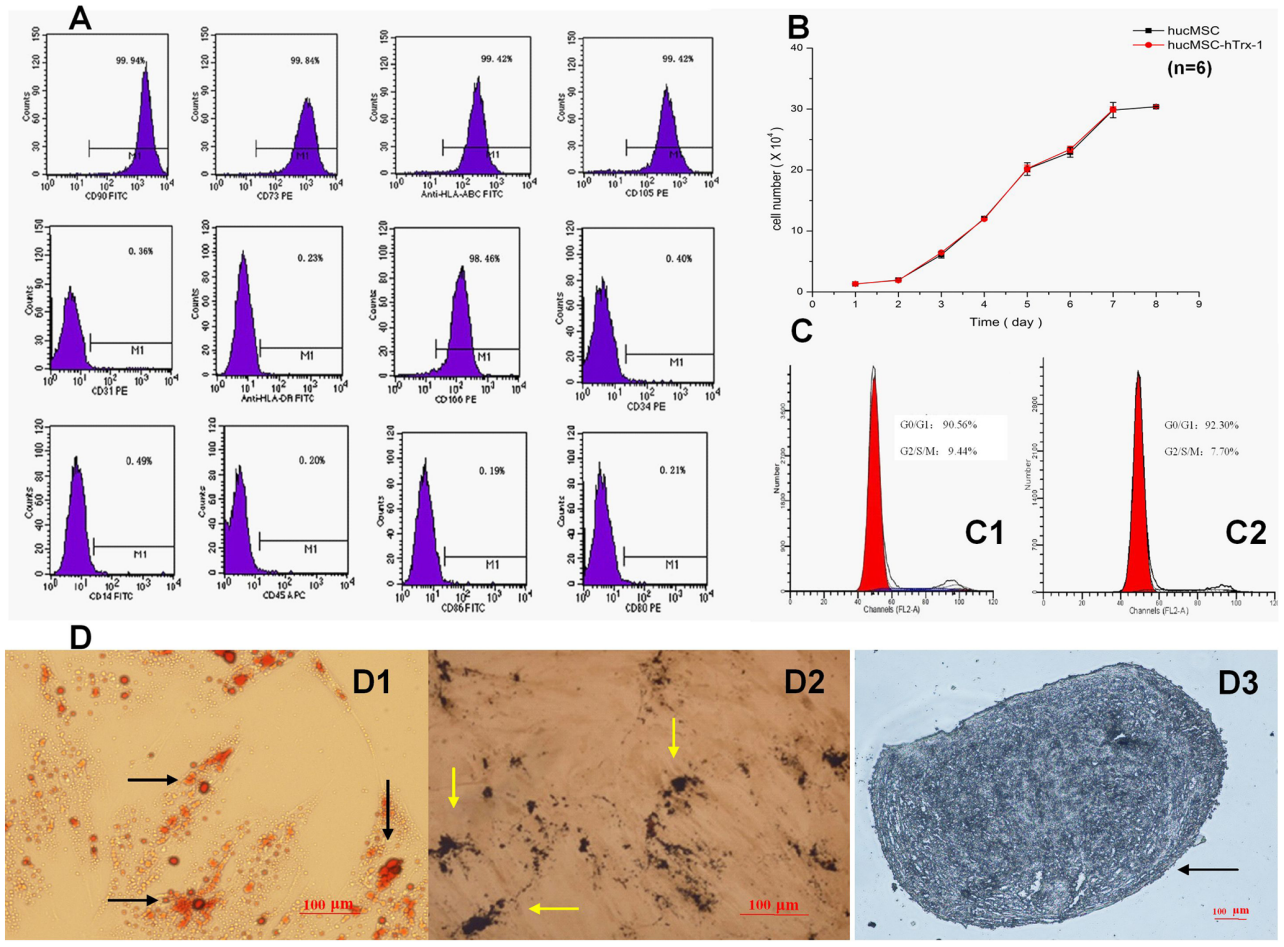
Biological characteristics of hucMSC-Trx-1. (A) hucMSC-Trx-1 retained the immunophenotypic features of hucMSC (positive markers: HLA-I, CD73, CD90, CD105 and CD166; negative markers: HLA-II, CD14, CD31, CD34, CD45, CD80 and CD86). (B) The growth curves of hucMSC and hucMSC-Trx-1. Both curves appeared in “S” shape and their population doubling times had no significant difference (n = 3, P>0.05). (C) A similar G0/G1 cell cycle status were observed in the hucMSC-Trx-1 group (92.30%) and hucMSC group (90.56%). (D) hucMSC-Trx-1 were induced to differentiate into lipoblasts on day 14 (D1), osteogenesis on day 21 (D2) and chondrogenesis on day 20 (D3). Arrows indicate the lipid droplets with orange-red (Oil Red-O staining, ×100), the calcium deposition with black granules (von kossa stain, ×100) and the microsphere with mazarine (toluidine blue stain, ×100).

The third generation of infected hucMSC showed adhesive growth without an obviously abnormal phenotype (Fig. 2 A) or proliferation profile. The growth curves for both hucMSC and hucMSC-Trx-1 showed an “S” shape, with similar population doubling times (Fig. 2 B). The proportion of hucMSC-Trx-1 and non-infected cells in G0/G1 phase were 93.21% and 90.56% respectively (Fig. 2 C).

As was the case for normal hucMSC, hucMSC-Trx-1 expressed high levels of the HLA-I antigens, CD105, CD73, CD90, CD166, and CD106. Both types of cells expressed low or undetectable levels of hematopoietic cell markers (CD14, CD34 and CD45) and allograft rejection-associated surface markers such as HLA-II antigens, CD80, and CD86 (Fig. 2 A). Similarly, hucMSC-Trx-1 cells differentiated into adipose, osteoid, and pseudocartilage cells in their respective induction media (Fig. 2D). After seven days of osteogenic induction, the hucMSC-Trx-1 morphology was mostly transformed from a spindle shape into polygons and cubes; 20 days after osteogenic induction, refractive particles were observed, and calcium deposition was observed as black granules by von Kossa staining (Fig. 2 D2). After 10 days of adipogenic induction, a small amount of cytoplasm vacuolization was observed. Lipid droplets were clearly observed 14 days later by oil red O staining (Fig. 2 D1). The microsphere of cartilage were formed on 20 days after chondrogenesis induction (Fig. 2 D3).

### hucMSC-Trx-1 injection enhances redox capacity in vivo

**Figure 3:**
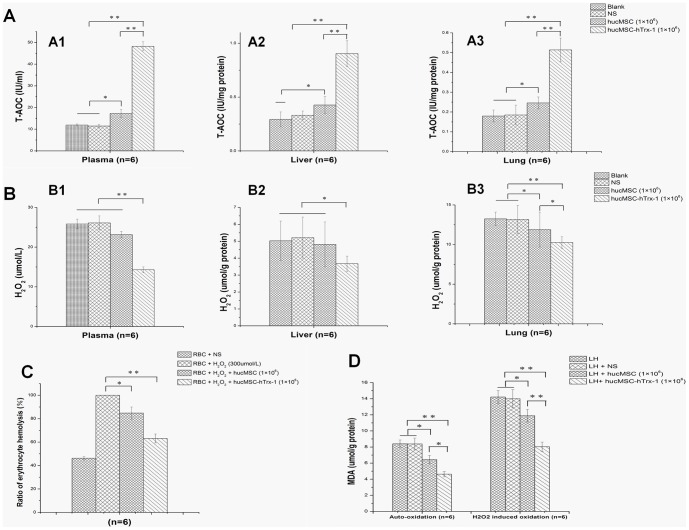
Redox capacity of hucMSC-Trx-1. A-B NOD/SCID mice (23.0±1.0 g) were randomly divided into four groups: hucMSC-Trx-1, hucMSC, NS and untreated group. The two former groups were injected *i.v.* 0.2 ml NS mixed with 1×10^6^ hucMSC-Trx-1 and 1×10^6^ hucMSC, respectively, the third group was injected *i.v.* 0.2 ml NS alone and the last group was without treatment as blank control. Mice (n = 6 in each group) were sacrificed at 48 hours after injection to obtain plasma, lung and liver tissue which were used for subsequent colorimetric determination of T-AOC and H_2_O_2_. (A1-3) In vivo effect of hucMSC-Trx-1 on T-AOC (hucMSC-Trx-1 *vs.* hucMSC or NS, ^**^ *P*<0.01). (B1-3) *In vivo* effect of hucMSC-Trx-1 on H_2_O_2_ content (hucMSC-Trx-1 *vs.* NS in plasma and lung, ^**^ *P*<0.01; hucMSC-Trx-1 *vs.* NS in liver, ^*^ *P*<0.05; hucMSC-Trx-1 *vs.* hucMSC in lung, ^*^ *P*<0.05). C-D the RBC and liver homogenate obtained from mice (n = 6 in each group) were co-cultured with 1×10^6^ hucMSC-Trx-1, 1×10^6^ hucMSC or NS for 1 hour, and then attacked by 300 µM H_2_O_2_ in the next hour. (C) *In vitro* Effect of hucMSC-Trx-1 on H_2_O_2_-induced RBC hemolysis. (hucMSC-Trx-1 *vs*. NS, ^**^ *P*<0.01; hucMSC-Trx-1 *vs.* hucMSC, ^*^ *P*<0.05). (D) *In vitro* effect of hucMSC-Trx-1 on liver homogenate lipid peroxidation. Auto-oxidation (hucMSC-Trx-1 *vs.* NS or untreated, ^**^ *P*<0.01; hucMSC-Trx-1 *vs.* hucMSC, ^*^ *P*<0.05). H_2_O_2_-induced oxidation (hucMSC-Trx-1 *vs.* hucMSC or NS or untreated, ^**^ *P*<0.01).

Next, we tested the redox capacity of blank group, NS group, hucMSC group and hucMSC-Trx-1 group by measuring T-AOC and H_2_O_2_ concentrations after the injection of saline or cell suspensions in mice. As shown in Fig. 3 A and B, 1×10^6^ hucMSC-Trx-1 improved the total antioxidant capacity of the plasma, liver, and lung tissues, while no such improvement was observed in the NS and hucMSC groups (p<0.01). Meanwhile, Trx-1 reduced endogenous H_2_O_2_ more effectively and more rapidly than the NS (plasma and lung, p<0.01; liver, p<0.05) and hucMSC treatments (lung, p<0.05; plasma and liver, no significant difference).

### hucMSC-Trx-1 prevents oxidative damage in vitro

H_2_O_2_-induced oxidative damage is first manifested in the destruction of the membranous structure of cells or membrane-bound organelles. In the RBC hemolysis assay (Fig. 3 C), H_2_O_2_ damaged the membrane of murine red blood cells and led to hemolysis. The hemolysis ratio of the oxidation group, which was exposed to 300 µM H_2_O_2_ alone, was significantly higher (defined as 100%) (p<0.01) than that of NS group. However, the hemolysis ratio was significantly lower when the erythrocytes were co-cultured for one hour with 1×10^6^ hucMSC-Trx-1 prior to H_2_O_2_, and this effect was stronger than that produced by hucMSC (p<0.05).

MDA is produced from the lipid peroxidation of a membrane structure. *In vitro*, the liver tissue homogenate had increased MDA content due either to auto-oxidation or H_2_O_2_-induced oxidation. However, in the presence of hucMSC-Trx-1 pre-protection, the MDA from both oxidation modes decreased significantly (p<0.01). For H_2_O_2_-induced oxidation in particular, the anti-oxidation effect of hucMSC-Trx-1 was more efficient than that of hucMSC (p<0.01).

### The excessive inflammatory response is effectively reduced by hucMSC-Trx-1 in the lung, liver and intestine

**Figure 4:**
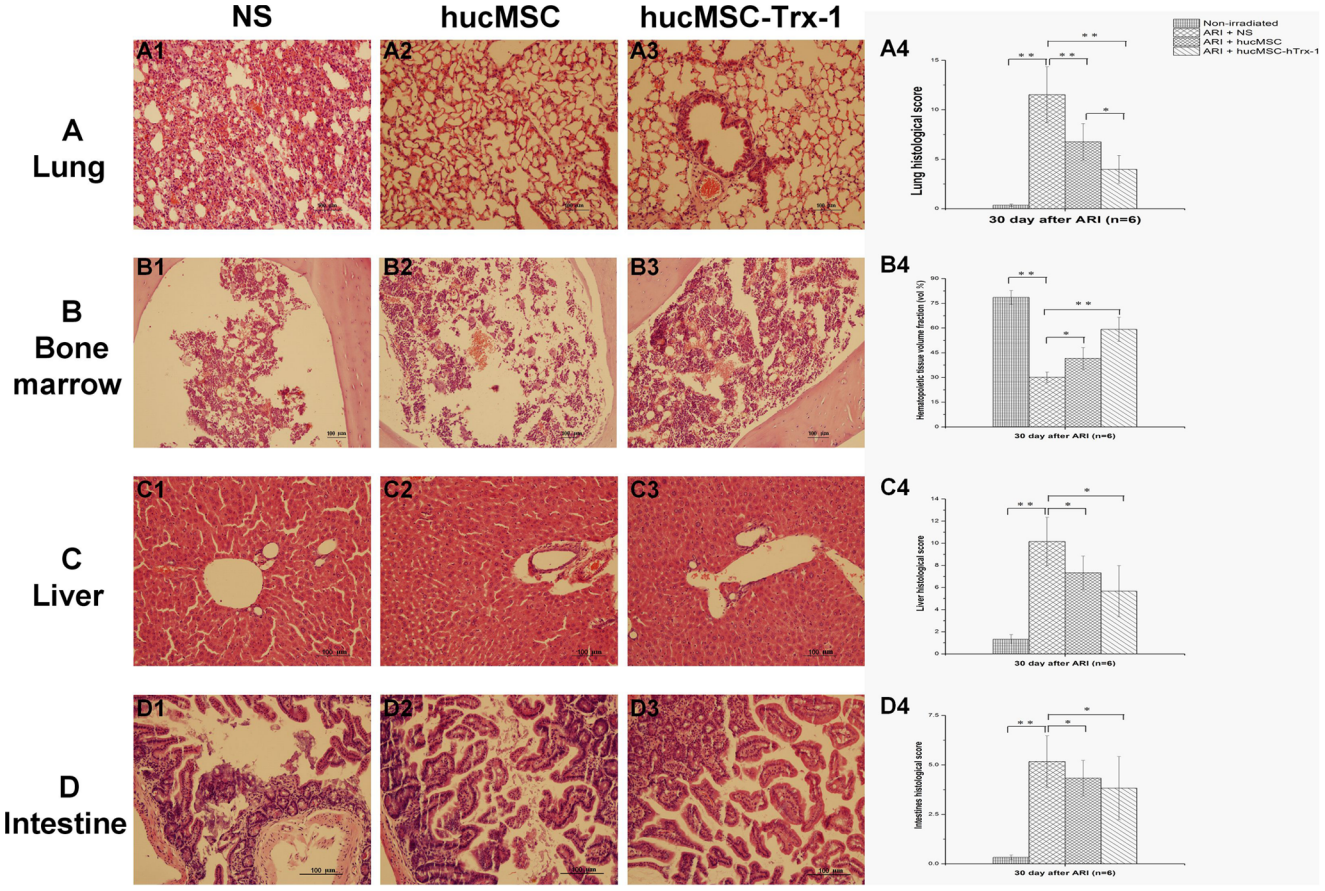
Pathology detection of the radio-sensitive tissues on day 30 after irradiation induced injury. On day 30, mice (n = 6 in each group) were randomly sacrificed to obtain femur, lung, liver and intestinal tissues for H.E. stain. The pathological sections were observed and estimated histological score by two independent pathologists blinded to the source of treatment. (A) Degree of inflammatory damage in lung. Compared with the other two groups, the mice of hucMSC-Trx-1 group had normal alveolar wall intervals, little vascular congestion and inflammatory cell infiltrations according to Underwood histological score (hucMSC-Trx-1 *vs.* NS, ^**^ *P*<0.01; hucMSC-Trx-1 *vs.* hucMSC, ^*^ *P*<0.05. ×100). (B) Degree of inflammatory damage in bone marrow. hucMSC-Trx-1 effectively protected the proliferation of the bone marrow after radiation according to HV/TV (hucMSC-Trx-1 *vs.* NS, ^**^ *P*<0.01; hucMSC-Trx-1 *vs.* hucMSC, ^*^ *P*<0.05. ×100). (C–D) Degree of inflammatory damage in liver and intestine. The effects of hucMSC-Trx-1 on inhibition of inflammation progression and decrease of tissue damage in liver or intestine are more efficient than that of hucMSC and NS according to Kamal/Obermeier histological score (hucMSC-Trx-1 *vs.* NS, ^*^ *P*<0.05; hucMSC-Trx-1 *vs.* hucMSC, ^*^ *P*<0.05. ×100).

The damage in NS group became progressively more severe over time. Significant damage was observed in the bone marrow and lung (p<0.01) (Fig. 4 A4 B4), while in the liver and intestine, the pathological changes were relatively mild (though still significant, p<0.05) (Fig. 4 C4 D4). In pathological sections, we measured the hematopoietic tissue capacity in the bone marrow; NS group showed a sharp drop in capacity (Fig. 4 B1), while that of hucMSC group was only slightly reduced (Fig. 4 B2); hucMSC-Trx-1 group maintained a state of active hematopoiesis (Fig. 4 B3). Thirty days after treatment, histopathology showed that NS group had significantly widened alveolar walls and vascular congestion, hemorrhage, interstitial edema, and hyaline membrane formation in the lung tissue (Fig. 4 A1). In hucMSC group, widened alveolar walls with vascular congestion were also observed, with a moderate amount of inflammatory cell infiltration (Fig. 4 A2); hucMSC-Trx-1 group had a significantly lower histological score than the other two groups (p<0.01) (Fig. 4 A4), with normal alveolar wall intervals, only slight vascular congestion, and slight infiltration of inflammatory cells (Fig. 4 A3). Meanwhile, the liver tissue from the NS group showed mostly piecemeal or bridging necrosis, with obvious inflammation in the portal area (Fig. 4 C1); hucMSC group showed mostly spotty necrosis and focal inflammation (Fig. 4 C2); hucMSC-Trx-1 group had only slight necrosis and inflammation (Fig. 4 C3). hucMSC-Trx-1 group had significantly lower histological scores than the other two groups (p<0.05) (Fig. 4 C4). Similarly, the intestinal pathological sections in hucMSC-Trx-1 group showed less injury to the epithelium and less infiltration of inflammatory cells (p<0.05) than was observed in the other two groups (Fig. 4 D).

### hucMSC-Trx-1 infusion enhances the protection of the bone marrow hematopoietic system and promotes hematopoietic stem cell recovery after acute radiation injury

**Figure 5:**
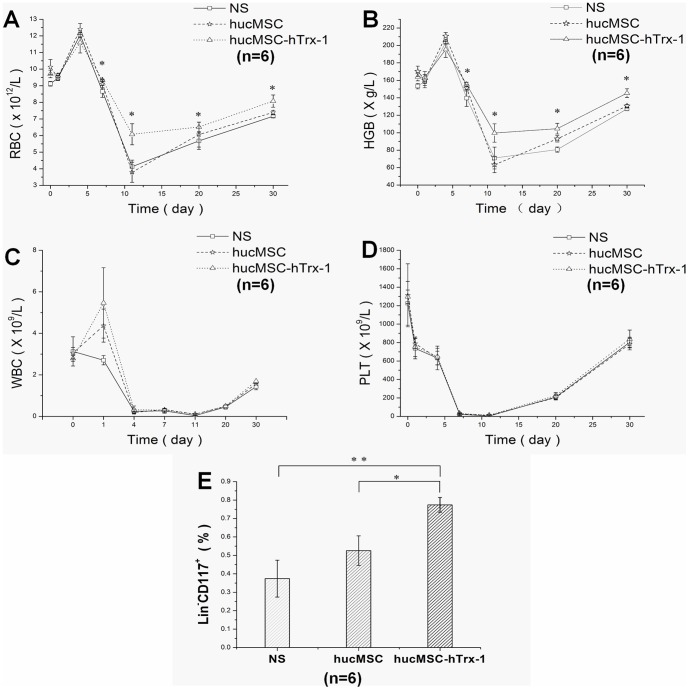
Radioprotective effect of hucMSC-Trx-1 on hematopoietic system. On day 1, 4, 7, 11, 20 and 30, 20 µl tail vein blood of mice (n = 6 in each group) were collected for subsequent detection. (A-D) The changes of peripheral RBC, HGB, WBC and PLT in different groups. After radiation injury, the WBC in the mice of each group showed a sharp decline, but the recovery erythrocyte and hemoglobin content in hucMSC-Trx-1 group mice was significantly higher than those in hucMSC group and NS group (hucMSC-Trx-1 *vs.* hucMSC or NS, ^*^ *P*<0.05). (E) Effect of hucMSC-Trx-1 on hematopoietic stem cells. On day 30, hucMSC-Trx-1 group had a significantly higher ratio of bone marrow Lin^−^CD117^+^ cells than hucMSC group and NS group (hucMSC-Trx-1 *vs.* hucMSC, ^*^ *P*<0.05; hucMSC-Trx-1 *vs.* NS, ^**^ *P*<0.01).

Routine blood tests showed that levels of hemoglobin, erythrocytes, and leukocytes transiently increased in the first four days after radiation injury and then began to decline. In contrast, platelets declined from the beginning. The erythrocyte and hemoglobin contents in hucMSC-Trx-1group mice were significantly higher than NS group mice (p<0.05) at 7 d, 11 d, 20 d, and 30 d; furthermore, these levels were significantly higher in hucMSC-Trx-1 group than hucMSC group (p <0.05) at 11 d, 20 d, and 30 d. The erythrocyte and hemoglobin contents in hucMSC group mice were not significantly higher than in NS group from 7 d to 30 d (Fig. 5 A, B). In hucMSC-Trx-1 group, leukocyte and platelet recovery was slightly better than in NS group and hucMSC group, but there were no significant differences among the three groups (Fig. 5 C, D).

The flow cytometry results examining the bone marrow Lin^−^CD117^+^ cell ratio at 30 days in each group showed that hucMSC-Trx-1 group had a significantly higher ratio of bone marrow Lin^−^CD117^+^ cells than hucMSC group and NS group (hucMSC-Trx-1 *vs*. hucMSC, *P*<0.05;hucMSC-Trx-1 *vs*. NS, *P*<0.01) (Fig. 5 E).

### hucMSC-Trx-1 infusion improves the quality of life and prolongs survival

**Figure 6:**
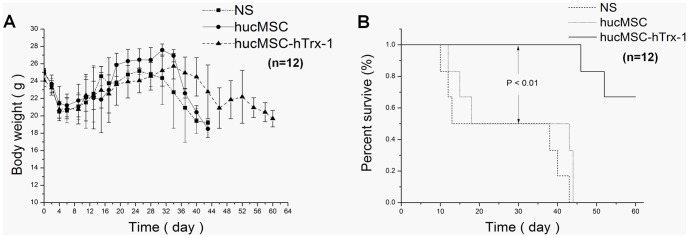
The effects of hucMSC-Trx-1 on body weight change and survival time of mice after ARI. After ARI treatment, mice (n = 12 in each group) were observed and recorded daily for the number of survivals, change of weight, and state of activity. (A) Changes of body weight. In the early days after ARI, the body weights of mice decreased rapidly in all three groups, and rapid weight decline appeared again on day 30∼32 in hucMSC group and NS group until all mice died. But in these days, a slowly declined body weight was observed in hucMSC-Trx-1 group and this trend did not cease until the end of the 60 day's observation. (B) Prolonged median survival time in hucMSC-Trx-1 group. The NS group (26±14.43 d) and hucMSC group (29.33±14.49 d) showed no significant difference (P = 0.36), but hucMSC-Trx-1 group (56.67±5.25 d) was significantly prolonged compared with hucMSC-Trx-1 group and NS group (hucMSC-Trx-1 *vs.* hucMSC or NS, *P*<0.01).

After the radiation injury, NS group animals showed listlessness, lethargy, reduced activity, and reduced food consumption; no diarrhea was observed, but mice began dying on day 10. At 14 d, the general situation had worsened, and all animals had died by day 43. The early symptoms of hucMSC group were similar to those of NS group; the mice started to die on day 12, and all mice had died by day 44; however, the recovery was slightly better in hucMSC group than in NS group. The early symptoms of hucMSC-Trx-1 group were the same as those of hucMSC group and NS group; however, on day 14, the general state of the mice in hucMSC-Trx-1 group was significantly better. The mice began to die on day 46. At 60 d, the survival rate remained 66.7%. During the early days following radiation injury, the body weight of mice decreased rapidly in all three groups; it decreased rapidly again on days 30–32 in hucMSC group and NS group until all mice had died. During the same period, a slow decline in body weight was detected in hucMSC-Trx-1 group until the end of the 60 -days observation period (Fig. 6 A).

The median survival times of mice from hucMSC group (26±14.43 d) and NS group (29.33±14.49 d) were similar (p = 0.36); survival in hucMSC-Trx-1 group (56.67±5.25 d) was significantly longer than in hucMSC group or NS group (p <0.01) (Fig. 6 B).

## Discussion

This study shows that the infusion of Trx-1-overexpressing hucMSC can prolong survival in mice with ARI. At the same time, we confirmed that hucMSC-Trx-1 can enhance the body's redox capacity, prevent damage caused by free radicals, and reduce excessive inflammatory responses in important organs. In addition, hucMSC-Trx-1 adenovirus-mediated infection can produce high Trx-1 gene expression without changing the biological characteristics (proliferation, growth cycle, phenotype, differentiation capacity, and low immunogenicity) of the hucMSC.

ARI not only causes direct DNA damage to tissues [39], [40], it also produces many active small molecules that cause lipid peroxidation damage, increased inflammatory response, and the inhibition of DNA recovery [41]-[43]. This might be of particular relevance to treating irreversible damage in important organs and tissues.

Survival rate and quality of life are both impaired by ARI [44], [45]. We observed a survival rate of 66.7% in the hucMSC-Trx-1 group; on the other hand, the mice in the two other groups had all died by day 44; as a result, mice in HucMSC-Trx-1 group had experienced a 27-day improvement in median survival time (56.7±5.25 days in hucMSC-Trx-1 group *vs*. 29.3±14.49 days and 26±14.43 days in the two control groups). In addition, hucMSC-Trx-1 promoted a better quality of life (e.g., activity status, weight changes) than the other two treatments, especially 14 days after ARI.

The overall body changes that occur after radiation injury consist of multiple pathological effects in different organs. Certain important organs (bone marrow, lung, liver, and intestines) are more vulnerable to radiation because the ionizing radiation not only causes direct injury to these organs but also causes synergistic effects from bystander injury [46], [47] due to oxidative stress, defects in signal transduction, and changes to the cellular microenvironment. Upon exposure to radiation, the tissue components are activated or ionized, which leads to the breaking of chemical bonds, the generation of free radicals, and, eventually, a systemic inflammatory response syndrome (SIRS) [48]-[50]. Radiation also causes reduced levels of antioxidant enzymes and eventually leads to DNA and cell membrane damage, the inactivation of important enzymes and proteins and, finally, cell breakdown or apoptosis. RBCs have no organelles; instead, they contain a metal-complexing protein (hemoglobin) that induces and initiates lipid peroxidation. The plasma membrane is rich in polyunsaturated fatty acids. Therefore, the erythrocyte cell membrane is susceptible to attack by free radicals and consequent lipid peroxidation and hemolysis. Liver tissue homogenate contains many membrane-bound organelles. Although they are partially protected by their membranes, these organelles are susceptible to oxidative damage, which leads to lipid over-oxidation followed by the generation of a large number of lipid superoxide products (e.g., MDA). Both erythrocytes and liver tissue homogenate can be used for models of free radical damage on membranes and membrane-bound organelles. Our results indicate that hucMSC-Trx-1 greatly decreased the generation of MDA in murine liver tissue homogenate and decreased RBC hemolysis in cases of H_2_O_2_-induced damage. The infusion of hucMSC-Trx-1 into mice improved the T-AOC of plasma, the liver, and lung and improved the free radical scavenging functions of these organs, indicating that hucMSC-Trx-1 has strong anti-oxidative properties.

Inflammatory stages and oxidative stress are closely associated. The imbalance of oxidation and antioxidant activity in the internal environment leads to tissue damage. Excessive reactive oxygen species can promote inflammatory responses and induce inflammatory cascade reactions, while slow inflammatory processes stimulate the generation of oxidative stress; thus, a vicious cycle is created that continues to aggravate the damage [51]. Our histopathological results show that hucMSC-Trx-1 improved the microenvironment of important organs, reducing excessive local inflammation reactions and promoting recovery from oxidative damage.

The destruction of the hematopoietic system has catastrophic consequences for the animal in the early stage of ARI. HSC, which mainly come from the bone marrow, play a decisive role in the formation of various blood cells in adult mammalian hematopoietic systems. However, HSC are extremely sensitive to radiation, as are murine bone marrow stem cells [52]. Under normal conditions, HSC can differentiate into various lineages and replace senescent hematopoietic cells. When exposed to high-dose radiation, the blood cells will significantly decrease in number, and the hematopoietic system will fail as soon as the HSC self-renewal ratio cannot maintain the necessary differentiation [53]. Because they do not express CD34 [54], murine HSC must be separated and identified by the combination of Lin^−^ and CD117^+^, which represent the immature cell lineage and pluripotent stem cells, respectively [55], [56]. We found that Lin^−^CD117^+^ cells existed in significantly different proportions in the hucMSC-Trx-1, hucMSC and control mice 30 days after irradiation. This indicates that, although hucMSCs can protect residual hematopoietic stem cells, hucMSC-Trx-1 enhanced this protective effect. The detected concentrations of RBCs, HGB, WBCs, and PLTs confirmed this conclusion.

Taken together, our results demonstrate that the combination therapy of hucMSC and Trx-1 (where Trx-1 is expressed in hucMSC by means of an adenovirus), is effective at protecting and recovering from ARI damage in NOD/SCID mice. This combination therapy also effectively reduces the excessive inflammatory response in important organs, helping these organs repair the ARI-induced damage. However, we should note that our conclusions are based only on observations in animals. The specific molecular mechanism of the radioprotective effect of this therapy is still not clear, and the optimal conditions for its practical application are not known. Therefore, further studies are warranted to investigate the source of its beneficial effects.

## Supporting Information

Figure S1 **Pathological observation of lung, bone marrow, liver and intestine on day 30 after irradiation induced injury.** On day 30, mice (n = 6 in each group) were randomly sacrificed to obtain femur, lung, liver and intestinal tissues for H.E. stain. (A) Pathological observation of lung, ×100. (B) Pathological observation of bone marrow, ×100. (C) Pathological observation of liver, ×100. (D) Pathological observation of intestine, ×100.
(TIF)
