# Supplementary figures and images for: Infusion of Trx-1-Overexpressing hucMSC Prolongs the Survival of Acutely Irradiated NOD/SCID Mice by Decreasing Excessive Inflammatory Injury
Source: PLoS One. 2013 Nov 4;8(11):e78227. doi: 10.1371/journal.pone.0078227 (PMC3817237; doi:10.1371/journal.pone.0078227)

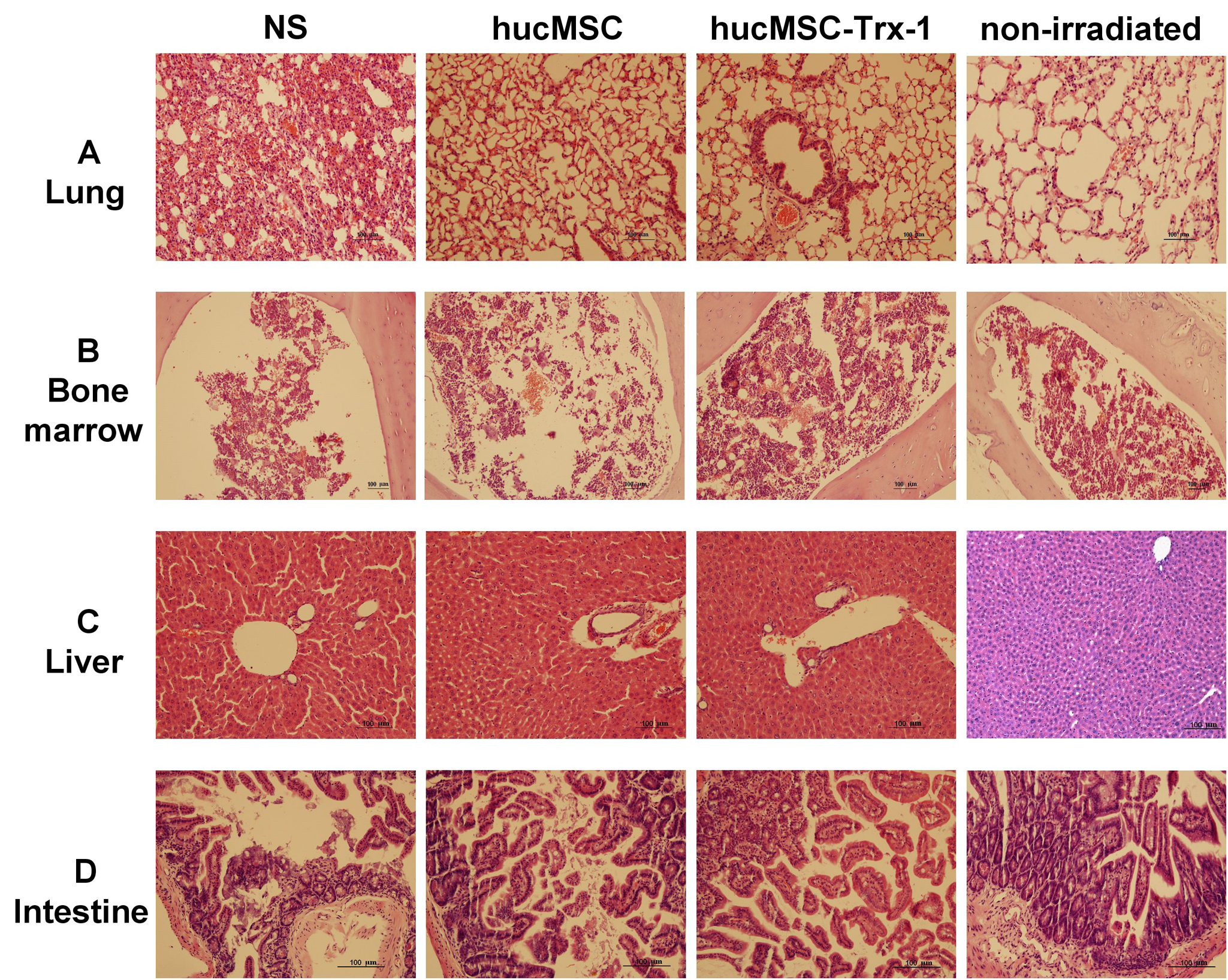

Supplement: Figure S1 — Pathological observation of lung, bone marrow, liver and intestine on day 30 after irradiation induced injury. On day 30, mice (n = 6 in each group) were randomly sacrificed to obtain femur, lung, liver and intestinal tissues for H.E. stain. (A) Pathological observation of lung, ×100. (B) Pathological observation of bone marrow, ×100. (C) Pathological observation of liver, ×100. (D) Pathological observation of intestine, ×100. (TIF) [file pone.0078227.s001.tif]
